# Ultrafiltration during continuous hemofiltration in stabilized ICU patients is not associated with microcirculatory perfusion derangements

**DOI:** 10.1186/cc10980

**Published:** 2012-03-20

**Authors:** B Scheenstra, G Veenstra, M Koopmans, WP Kingma, H Buter, HM Hemmelder, EC Boerma

**Affiliations:** 1 Medical Centre Leeuwrden, the Netherlands

## Introduction

Ultrafiltration during intermittent haemodialysis has been associated with reduction in microcirculatory perfusion, as observed with sidestream dark-field (SDF) imaging [1]. This technique has also been useful in the evaluation of volume status in critically ill patients [2]. To date no data are available on the influence of ultrafiltration during continuous venovenous hemofiltration (CVVH) on microcirculatory perfusion.

## Methods

In this single-centre, prospective, observational study patients with acute renal failure on CVVH were included after hemodynamic stabilization and written informed consent A fixed dose of net ultrafiltration was calculated for each patient, aiming at a negative total fluid balance of 50 ml/hour. Microcirculatory perfusion was observed with sublingual SDF imaging after 1 hour of zero balance CVVH (T1) and additionally after 1 hour of negative fluid balance ultrafiltration (T2). The primary outcome was a change in microvascular flow index (MFI) between T1 and T2. Data are presented as median (IQR). Differences are calculated with a nonparametric test for paired data.

## Results

Eleven patients were eligible for the study; one denied informed consent. One patient could not be evaluated due to the unavailability of the research team and in two patients we were unable to obtain images of proper quality. The median APACHE II score was 26 (21 to 29); at baseline LOS ICU was 5 (3 to 6) days and fluid balance +7.9 (5.1 to 14.2) l. Hemodynamic and microcirculatory variables are depicted in Table 1.

**Table 1:** (Micro)circulatory variables during ultrafiltration

	T1	T2	*P *value
RR mean	71 (65 to 94)	66 (63 to 95)	0.87
Heart rate	97 (78 to 126)	94 (75 to 123)	0.03
MFI	2.9 (2.7 to 3)	3 (3 to 3]	0.34
TVD	20 (18 to 22)	21 (17 to 23)	0.5

## Conclusion

A negative net fluid balance of 50 ml/hour during ultrafiltration in CVVH is not associated with microcirculatory perfusion alterations.
